# Can AI replace psychotherapists? Exploring the future of mental health care

**DOI:** 10.3389/fpsyt.2024.1444382

**Published:** 2024-10-31

**Authors:** Zhihui Zhang, Jing Wang

**Affiliations:** ^1^ College of Landscape and Horticulture, Yunnan Agricultural University, Kunming, China; ^2^ Barcelona School of Architecture, Universitat Politècnica de Catalunya, Barcelona, Spain; ^3^ Department of Ultrasound, Shenzhen Second People’s Hospital, Shenzhen, China

**Keywords:** Artificial Intelligence (AI), psychotherapy, algorithmic bias, mental health care, ethical considerations

## 1 Introduction

**Figure 1:**
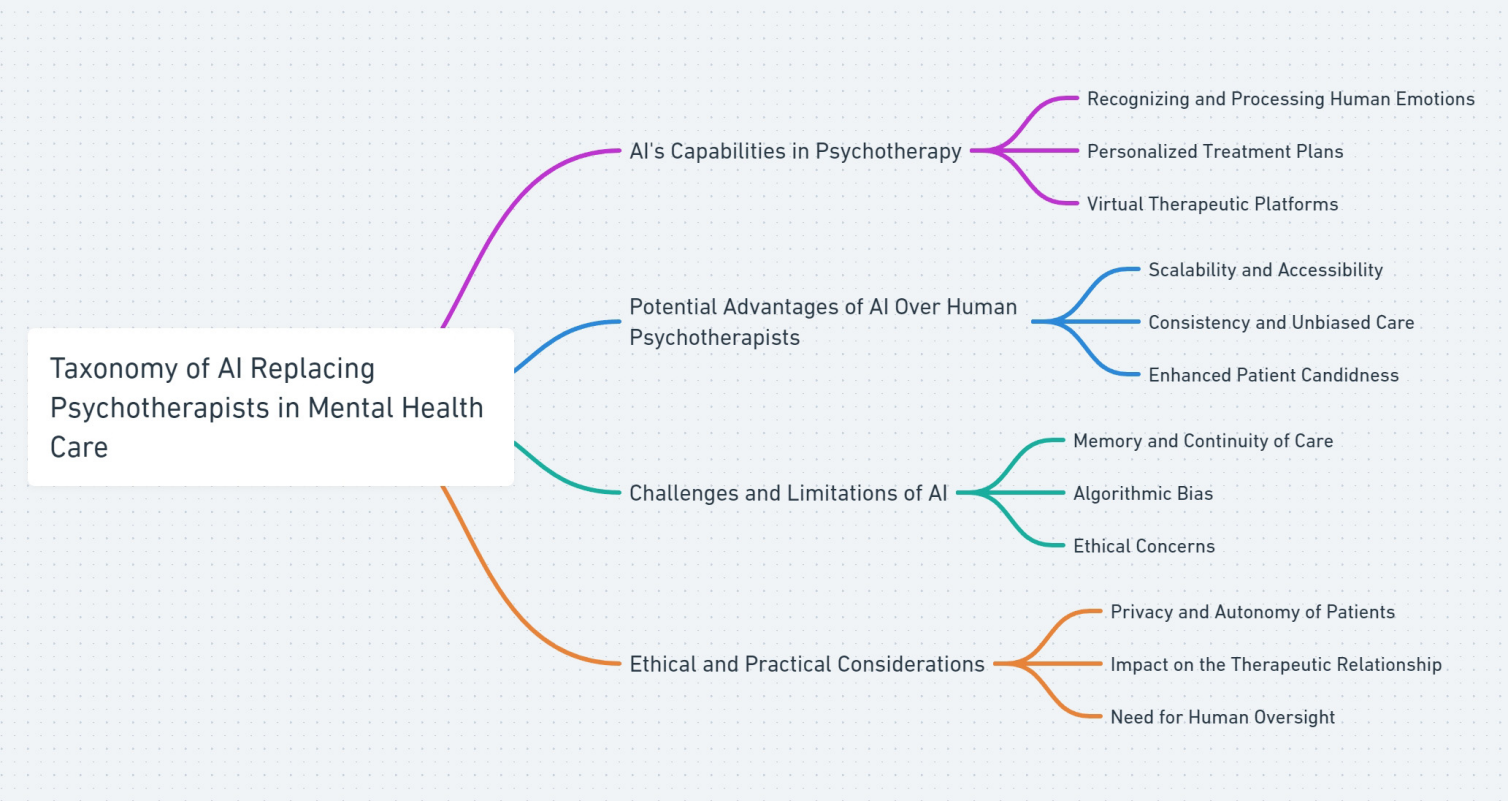
Taxonomy of AI’s role in replacing psychotherapists, highlighting AI’s ability to recognize human emotions, potential advantages, challenges, and ethical considerations in mental health care.

In the current technological era, Artificial Intelligence (AI) has transformed operations across numerous sectors, enhancing everything from manufacturing automation to intelligent decision support systems in financial services (1, 2). In the health sector, particularly, AI has not only refined the accuracy of disease diagnoses (3) but has also ushered in groundbreaking advancements in personalized medicine (4). The mental health field, amid a global crisis characterized by increasing demand and insufficient resources, is witnessing a significant paradigm shift facilitated by AI, presenting novel approaches that promise to reshape traditional mental health care models (5, 6) (see **Figure 1**).

Mental health, once a stigmatized aspect of health care, is now recognized as a critical component of overall well-being, with disorders such as depression becoming leading causes of global disability (WHO). Traditional mental health care, reliant on in-person consultations, is increasingly perceived as inadequate against the growing prevalence of mental health issues (7, 8). AI’s role in mental health care is multifaceted, encompassing predictive analytics, therapeutic interventions, clinician support tools, and patient monitoring systems (9). For instance, AI algorithms are increasingly used to predict treatment outcomes by analyzing patient data (10). Meanwhile, AI-powered interventions, such as virtual reality exposure therapy and chatbot-delivered cognitive behavioral therapy, are being explored, though they are at varying stages of validation (11, 12). Each of these applications is evolving at its own pace, influenced by technological advancements and the need for rigorous clinical validation (13, 14).

Furthermore, the role of AI in mental health extends to potentially replacing certain functions traditionally performed by human psychotherapists. Innovations in machine learning and natural language processing have enabled AI systems like ChatGPT to recognize and process complex human emotions, facilitating interactions that once required the nuanced understanding of trained therapists (16, 17). Preliminary studies suggest that AI-powered chatbots may help alleviate symptoms of anxiety and depression (18–20). However, these studies often involve small participant groups and lack long-term follow-up, making it difficult to draw definitive conclusions about their effectiveness. Consequently, while AI interventions hold promise, further research through large-scale, randomized controlled trials is necessary to establish their efficacy and sustainability over time.

As AI continues to evolve and become more deeply integrated into the mental healthcare sector, its potential to fundamentally transform the field is undeniable. At a time when mental health issues have reached pandemic proportions globally, affecting productivity and quality of life (23, 24), the need for innovative solutions is urgent. AI’s integration into mental health services offers promising avenues for enhancing care delivery and improving treatment efficacy and efficiency. However, it is crucial to approach this evolution with caution. We must carefully address the limitations of AI, such as algorithmic bias, ethical concerns, and the need for human oversight, to prevent future disparities and ensure that AI complements rather than replaces the essential human elements of psychotherapy. This balanced approach will be key to harnessing AI’s benefits while safeguarding the quality and accessibility of mental health care.

## 2 AI’s capabilities and progress in psychotherapy

The integration of Artificial Intelligence (AI) in Psychotherapy represents a significant phase in the evolution of mental health care, leveraging technology to enhance both treatment efficacy and accessibility. Initial experiments in the 1960s, notably with the ELIZA program, showcased AI’s potential for therapeutic applications by mimicking human-like conversations (25). This pioneering work established the foundation for AI’s increasing role in psychological therapy and assessment over the ensuing decades.

The development of AI systems in the 1980s aimed to replicate human psychological expertise, leading to advanced diagnostic and therapeutic tools across various psychological disciplines (26, 27). By the end of the 20th century, this evolution gave rise to computerized cognitive behavioral therapy (CBT) programs, which were designed to provide structured, evidence-based interventions for common mental health conditions (28). Although these early applications were more basic than current AI technologies, they marked a pivotal shift toward enhancing the accessibility of mental health services through digital means (28). As technology advanced, the role of AI in mental health care rapidly expanded to encompass early detection of mental health issues, the creation of personalized treatment plans, and the introduction of virtual therapists and teletherapy enhancements (4, 15).

Continuing advancements in AI technology, driven by increases in computational power and breakthroughs in machine learning and natural language processing (NLP), have enabled more sophisticated interactions between AI systems and users. AI models, particularly those utilizing the transformer architecture like OpenAI’s ChatGPT (Version GPT-4o), demonstrate an exceptional capacity for recognizing the complexities of human emotion and language nuances (17). These models facilitate engaging conversations that adeptly interpret emotional states, providing contextually and emotionally relevant response (29). Future enhancements are expected to introduce more natural, real-time voice interactions and enable conversations with ChatGPT through real-time video, broadening the potential applications of AI in Psychotherapy (30). The upcoming GPT-5 is anticipated to surpass the current capabilities of GPT-4, further augmenting the effectiveness of AI applications in mental health care and extending the range of services available to patients (31).

### 2.1 ChatGPT in emotional awareness and mental health

A study by Elyoseph utilized the Levels of Emotional Awareness Scale (LEAS) to assess ChatGPT’s ability to recognize and articulate emotions in hypothetical scenarios (16). The findings indicate that ChatGPT can generate responses that reflect a level of emotional awareness similar to the general population. However, it’s important to note that ChatGPT’s ‘understanding’ is based on pattern recognition and language modeling, not on genuine emotional comprehension. Therefore, while ChatGPT can mimic emotional responses, it does not experience emotions as humans do, and its capabilities in this area are limited to textual analysis and generation.

### 2.2 Applications in mental health interventions

This proficiency in emotional comprehension positions ChatGPT as a potentially valuable tool in the field of psychotherapy and mental health care. For example, ChatGPT has been involved in pilot studies where it assisted in identifying early warning signs of mental health issues such as suicidal tendencies and providing initial therapeutic interventions (32). Moreover, recent research has demonstrated that general-purpose AI models, like Gemini Pro and GPT-4, outperform traditional therapeutic bots, such as Wysa and Youper, particularly in correcting cognitive biases like overtrust, fundamental attribution error, and the just-world hypothesis. In these areas, GPT-4 scored the highest, while therapeutic bots like Wysa scored the lowest (33).

### 2.3 Practical application: a hypothetical scenario

To illustrate how ChatGPT-4 can function in a therapeutic context, consider the following hypothetical scenario involving an individual seeking support from an AI-powered platform.

Practical Application of ChatGPT-4 in Psychotherapy

Alex is experiencing symptoms of anxiety related to work stress. Unable to access immediate professional help, Alex turns to a mental health app powered by ChatGPT-4 for support.

Alex: “I’ve been feeling really overwhelmed with work lately. Deadlines are piling up, and I can’t seem to catch up.”

ChatGPT-4: “I’m sorry to hear that you’re feeling this way. It sounds like you’re under a lot of pressure. Would you like to talk more about what’s causing these feelings, or perhaps explore some strategies to manage your stress?”

Alex: “I just feel like no matter how much I do, it’s never enough. I’m worried I’m going to let everyone down.”

ChatGPT-4: “It seems you’re experiencing a fear of disappointing others, which can be quite stressful. Remember, it’s important to acknowledge your efforts. Would discussing some time management techniques or ways to set realistic expectations be helpful?”

In this interaction, ChatGPT-4 provides empathetic responses, validates Alex’s feelings, and offers to explore coping strategies. This example demonstrates how AI-driven support can offer immediate assistance, especially for individuals who may lack access to mental health professionals or prefer the anonymity and convenience of a digital platform.

### 2.4 Implications for future psychotherapeutic practice

The integration of Artificial Intelligence (AI) in Psychotherapy promises transformative advancements in mental health care, each highlighted by recent research. Cunzhou Ran’s work on emotion analysis using ChatGPT demonstrates AI’s ability to recognize emotional nuances in communication, indicating potential for more empathetic interactions in therapy (34). Graham explores AI’s use in diagnosing and treating a range of mental health disorders, suggesting enhanced accuracy and efficacy in treatment plans (35). Saadia Gabriel’s examination of Large Language Models in clinical settings reveals AI’s capacity to broaden access to mental health services (36), though it emphasizes the need for stringent ethical standards. Lastly, Gilmar Gutierrez’s review on online mental healthcare underscores AI’s role in improving treatment adherence and patient engagement through continuous monitoring and support (37). Collectively, these studies suggest that while AI can significantly augment psychotherapeutic practice, careful consideration of ethical implications is essential.

## 3 Limitations of human psychotherapists

### 3.1 Resource constraints

Human psychotherapists, while deeply committed to their practice, face significant constraints in terms of time and physical resources that impact their ability to manage large caseloads. The traditional model of psychotherapy, which involves one-on-one sessions lasting from thirty minutes to an hour, inherently limits the number of patients a therapist can see daily. This limitation becomes particularly acute in regions with a high demand for mental health services but a limited number of available professionals. The scarcity of resources can lead to increased wait times for patients, potentially delaying critical interventions and exacerbating mental health conditions. Extended wait times and limited access to necessary care can result in deterioration of patient conditions, which poses serious challenges to mental health systems globally (38, 39).

### 3.2 Emotional labor and professional burnout

The work of psychotherapists involves intense emotional labor as therapists routinely engage with the emotional and psychological distress of their clients. This constant exposure to high-stress situations requires a substantial emotional investment and can lead to significant professional burnout. Symptoms of burnout among psychotherapists often manifest as emotional exhaustion, depersonalization, and a reduced sense of personal accomplishment, which not only impacts their personal health and job satisfaction but also affects their professional performance. Over time, this can result in reduced empathy and attentiveness—key components of effective therapy—thereby negatively impacting therapeutic outcomes and patient satisfaction. The emotional toll of psychotherapy can thus lead to a higher turnover among mental health professionals, further straining the system and impacting the quality of care provided to patients (40–42).

### 3.3 Limited scope for personalization

Personalization in therapy is crucial for its effectiveness, yet human therapists face inherent limitations in how extensively and accurately they can tailor their approaches to each individual patient. Despite their best efforts, human memory constraints and cognitive biases can affect a therapist’s ability to consistently integrate and recall detailed patient histories or subtle behavioral nuances across extended treatment periods. These limitations can hinder their capacity to fully personalize care, which is especially crucial for patients with complex, co-morbid conditions that require nuanced understanding and approach. In contrast, AI technologies, with their ability to process and remember vast amounts of information without bias, offer promising prospects for supporting more personalized and precise mental health interventions. AI can handle complex datasets, identify patterns, and recall details from patient interactions with greater accuracy than human therapists, potentially leading to improved treatment planning and outcomes (43–45).

## 4 Advantages of AI over human capabilities

### 4.1 Scalability and accessibility

AI significantly enhances the scalability and accessibility of mental health services. Unlike traditional therapy, which often requires physical presence and can be limited by geographic and resource constraints, AI-powered systems can provide support globally and operate continuously without fatigue. This accessibility is crucial, particularly in regions where mental health professionals are scarce (46, 47). Additionally, AI-driven interventions can reduce healthcare costs, making mental health care more affordable and accessible. The economic and operational benefits of AI in enhancing the reach of mental health services are well-documented (48).

### 4.2 Enhanced candidness in patient interaction

One of the unique advantages of AI in mental health is the increased candidness and openness that patients often exhibit when interacting with machines. Studies have shown that individuals are sometimes more willing to disclose sensitive information to AI systems due to perceived non-judgmental nature of machines (49, 50). This phenomenon can lead to more honest exchanges during therapy sessions, allowing for more accurate assessments and potentially more effective treatment. The absence of perceived judgment not only encourages more honest disclosures but also can reduce the stigma associated with seeking help for mental health issues, thus enhancing patient engagement (21).

### 4.3 Consistency and unbiased care

AI systems offer a level of consistency in mental health care delivery that human practitioners can find challenging to achieve due to natural variations in mood, fatigue, and personal bias. AI-driven tools apply the same standards and protocols to every interaction, ensuring all patients receive the same quality of care (51, 52). Furthermore, AI has the potential to reduce biases that can influence human judgment. These systems can be programmed to ignore irrelevant factors such as race, gender, or socio-economic status, promoting a more equitable healthcare environment (53).

## 5 Limitations of AI in psychotherapy

### 5.1 Memory

While AI systems like GPT-4 exhibit strong short-term memory capabilities, they still face significant challenges with long-term memory retention and integration. Current models often require external processes to summarize and track interactions over time, which limits their ability to maintain a continuous and coherent therapeutic relationship without human oversight (54–56). This limitation can affect the continuity of care, as AI may struggle to retain and integrate information from past sessions, potentially leading to fragmented or inconsistent therapeutic interactions. For effective psychotherapy, the ability to recall and integrate past information is crucial, and this remains a key area where human therapists currently hold an advantage over AI systems.

### 5.2 Bias

Algorithmic bias is a critical concern in the application of AI to mental health care. As highlighted by Akter, biases can arise from the data used to train AI models, leading to unequal treatment of individuals based on race, gender, or socioeconomic status, thus perpetuating existing disparities in mental health care (57). Despite efforts to minimize these biases, they are often unavoidable due to the decisions made during the development of AI systems. These biases can influence the interaction and effectiveness of AI in mental health settings, affecting how information is presented or what therapeutic approaches are emphasized. This reflects the inherent biases and priorities of the developers, which can have unintended consequences on the care provided to patients.

### 5.3 Long-term efficacy

While AI-based therapies have shown effectiveness in reducing symptoms of anxiety and depression in the short term, their long-term efficacy remains questionable. Studies have indicated that the initial benefits of AI-driven interventions often diminish over time, with no significant long-term improvements observed (22). This may be due to AI’s current inability to adapt to the evolving and complex nature of human mental health needs over extended periods. Unlike human therapists, who can adjust their therapeutic strategies based on ongoing interactions and deeper understanding of a patient’s history, AI systems may lack the flexibility required to maintain therapeutic effectiveness in the long term. This limitation highlights the need for continuous human oversight and potentially hybrid models where AI supports but does not replace the human element in psychotherapy.

### 5.4 Ethical considerations

Beyond technical limitations like memory and bias, the use of AI in mental health care raises significant ethical questions regarding privacy, autonomy, and the potential stigmatization of patients. As noted by Walsh, AI systems, particularly those that utilize biomarkers and other sensitive data, must navigate the complex balance between improving care and respecting patient privacy and autonomy (58). The design and implementation of AI in healthcare must prioritize ethical considerations, including strategies to mitigate bias, ensure transparency, and maintain patient trust (59). Without careful ethical oversight, AI could lead to unintended consequences such as misdiagnosis or the erosion of the therapeutic relationship between patients and human therapists. Additionally, the concept of precision psychiatry, which leverages AI to tailor interventions to individual needs, presents its own set of ethical challenges. Fusar-Poli et al. (2022) emphasize the need for human oversight to ensure that AI complements rather than replaces the nuanced understanding that human therapists bring to psychiatric care (60).

### 5.5 Comparison to human psychotherapists

Despite the advancements in AI technology, there are critical areas where AI falls short compared to human psychotherapists. Firstly, AI systems lack genuine empathy and the ability to form deep emotional connections with patients. Human therapists use their own emotional understanding to build trust and rapport, which is fundamental in therapy (61, 62). AI cannot authentically replicate this emotional resonance, as it does not experience emotions. Secondly, human therapists rely on professional intuition and ethical judgment to navigate complex therapeutic situations (63). They can interpret subtle cues and adapt their approach in real-time, something AI cannot do due to its reliance on predefined algorithms. Additionally, human therapists are adept at interpreting non-verbal communication, such as body language and facial expressions (64), providing deeper insight into a patient’s emotional state. AI systems, particularly those limited to text-based interactions, cannot perceive these cues. Moreover, cultural competence and sensitivity are areas where human therapists excel. They can tailor their approaches to align with a patient’s cultural background (65), whereas AI may not fully grasp cultural nuances, potentially leading to misunderstandings. Lastly, human therapists provide continuity of care and personalized treatment plans based on an evolving understanding of the patient’s history (66). AI may struggle with personalization to the same extent, potentially leading to less effective therapy experiences.

## 6 Conclusion

As artificial intelligence (AI) technology continues to advance, its increasing sophistication holds significant potential to reshape the landscape of mental health services. AI may play an important role in supporting psychotherapy, particularly in addressing the global mental health service gap where access to trained professionals is limited. By augmenting traditional mental health care, AI can offer scalable and cost-effective solutions that reduce barriers such as cost, stigma, and logistical challenges.

However, as AI becomes more integrated into mental health care, it is essential to recognize and address its limitations. Challenges related to memory retention, algorithmic bias, and ethical considerations underscore the need for a balanced approach. AI systems lack genuine empathy, ethical judgment, and the ability to interpret non-verbal cues—qualities that are intrinsic to human therapists. Therefore, these systems must be carefully designed and implemented to complement rather than replace the critical human elements of empathy, cultural competence, and nuanced understanding in therapy.

In this emerging paradigm, AI is envisioned not as a replacement for human therapists but as a powerful tool that extends the reach of mental health services. By automating routine tasks and providing support in areas where human professionals are scarce, AI can help ensure that individuals have greater access to essential mental health resources. Nevertheless, the integration of AI into mental health care must be pursued with caution, emphasizing fairness, transparency, respect for patient rights, and the necessity of human oversight. Ongoing research and rigorous clinical validation are crucial to establish the long-term efficacy and safety of AI interventions. When managed responsibly, this progression can be a pivotal step toward a more inclusive and effective global mental health strategy, blending technological innovation with the irreplaceable value of human connection.
